# Primary Pulmonary Myxoid Sarcoma Located in the Left Lung Parenchyma: Case Report with a Review of Literature

**DOI:** 10.70352/scrj.cr.24-0052

**Published:** 2025-04-09

**Authors:** Keisuke Hanawa, Toshihiko Soma, Tsuyoshi Shoji, Hiromichi Katakura

**Affiliations:** Department of Thoracic Surgery, Japanese Red Cross Otsu Hospital, Otsu, Shiga, Japan

**Keywords:** EWSR1:CREB1, FISH, lung tumor, primary pulmonary myxoid sarcoma, PPMS, wedge resection

## Abstract

**INTRODUCTION:**

Primary pulmonary myxoid sarcoma (PPMS) is a very rare low-grade sarcoma. It is known to have a characteristic chromosomal translocation at t(2;22)(q33;q12) and a distinctive genetic alteration, Ewing sarcoma breakpoint region 1 (EWSR1):cAMP response element binding protein 1 fusion. Most cases of PPMS reported so far have been found in the bronchi or bronchioles, and there are only a few cases of them arising from the peripheral lung parenchyma.

**CASE PRESENTATION:**

A 58-year-old man was referred to our department for diagnosis and treatment because a computed tomography (CT) scan showed a 15mm nodule in the left lung. For diagnosis and treatment, he underwent a video-assisted wedge resection. The tumor protruded from the lung parenchyma and had a very striking appearance. Histological features and immunostaining results were not enough to make the diagnosis. Fluorescence in situ hybridization (FISH) analysis was subsequently performed, which suggested EWSR1 gene rearrangement, leading to the final diagnosis of PPMS. The patient is alive 18 months postoperatively with no evidence of recurrence.

**CONCLUSIONS:**

We encountered a rare case of PPMS arising from the peripheral lung parenchyma. In addition, our case was diagnosed as an overlap lesion of PPMS and angiomatoid fibrous histiocytoma. We can expect a good prognosis with surgical resection alone for the treatment of PPMS, but more accumulation of cases is desired for the establishment of an accurate diagnosis and prediction of the disease course.

## Abbreviations

AFH
: angiomatoid fibrous histiocytoma
CT
: computed tomography
EMC
: extraskeletal myxoid chondrosarcoma
EWSR1
: Ewing sarcoma breakpoint region 1
FISH
: fluorescence in situ hybridization
H&E
: hematoxylin and eosin
IMT
: inflammatory myofibroblastomic tumor
PPMS
: primary pulmonary myxoid sarcoma
RT-PCR
: reverse transcription-polymerase chain reaction

## INTRODUCTION

Primary pulmonary myxoid sarcoma (PPMS) was newly added in the 4th edition of the World Health Organization classification in 2015,^1)^ and its first case was an endobronchial tumor reported by Thway et al. in 1999.^2)^ About 40 cases have been reported in the literature since then. Many cases are detected by close examination of clinical symptoms such as cough, hemoptysis, and chest pain, while some cases are asymptomatic and found incidentally. Although identification of the Ewing sarcoma breakpoint region 1:cAMP response element binding protein 1 (EWSR1:CREB1) fusion gene by reverse transcription-polymerase chain reaction (RT-PCR) is the most reliable method for diagnosis, in practice, PPMS is often diagnosed when EWSR1 gene rearrangement is found by fluorescence in situ hybridization (FISH) and the histological features and immunostaining results are consistent. We report here a rare case that occurred in the peripheral lung parenchyma with some discussion of the literature.

## CASE PRESENTATION

**Fig. 1:**
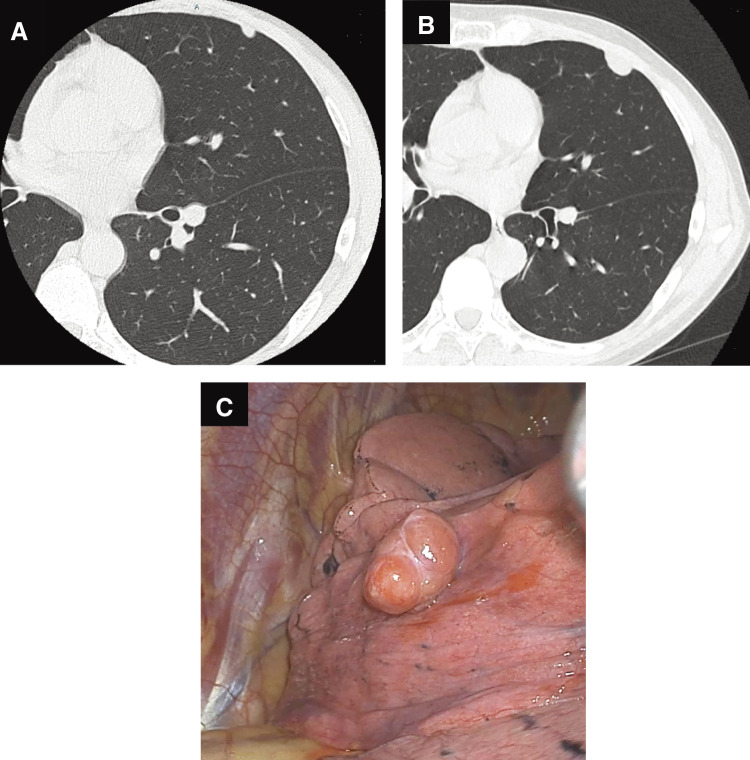
(**A**–**C**) CT images and intraoperative appearance of the lesion. (**A** and **B**) CT images showed that the tumor had clearly increased in size over the past 10 years. (**C**) The tumor was grossly protruding from the peripheral lung parenchyma with no implication of invasion into the surrounding tissue. CT, computed tomography

A 58-year-old man with a possible history of asbestos exposure was referred to our department because of an abnormal chest X-ray. His smoking history was 36 pack-years, and there was no significant family history. The results of a computed tomography (CT) revealed a solid 15 mm nodule just beneath the pleura in the lingual segment of the left lung. Since a CT scan taken at our hospital about 10 years ago showed a nodule in the same location and it was clearly enlarged, we decided to perform a surgical resection for diagnosis and treatment (**Fig. 1A** and **1B**). A video-assisted wedge resection was performed. Obvious pleural plaques were not observed in the chest cavity. Grossly, the tumor was clearly protruding from the peripheral lung parenchyma with no evidence of invasion into the surrounding tissue (**Fig. 1C**). The intraoperative pathological diagnosis was suspicious for mesothelioma.

**Fig. 2:**
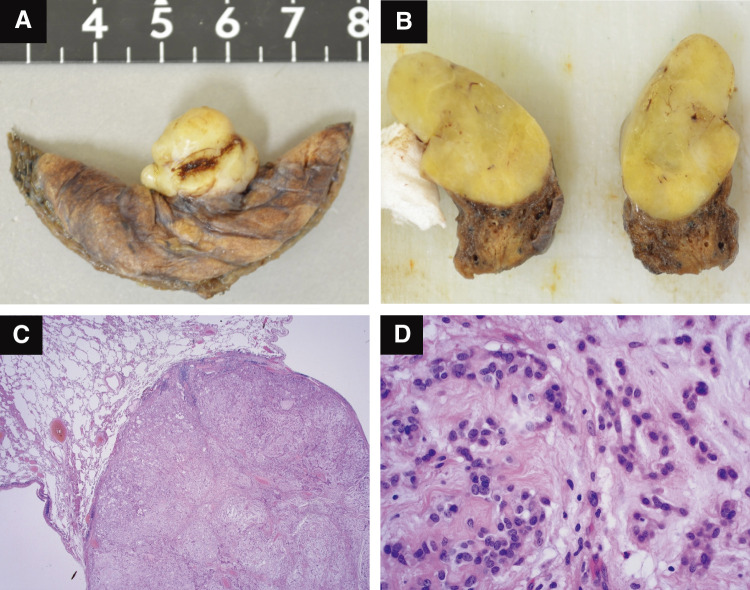
(**A**–**D**) Appearance of resected lesion and histology with H&E stain. (**A** and **B**) The lesion was 1.3 cm in diameter, arising in the peripheral lung and protruding outside, and the tumor was well-circumscribed and surrounded by a fibrous capsule. (**C** and **D**) It was multinodular and lobulated, and the stroma consisted of a mucus matrix with a reticulated growth of star- to spindle-shaped tumor cells (H&E Cx 20, Dx 200). H&E, hematoxylin and eosin

Histopathologically, the lesion was 1.3 cm in diameter, arising in the peripheral lung and protruding outside, and the tumor was well-circumscribed surrounded by a fibrous capsule. Asbestos bodies were not detected in the resected lung tissue. The tumor was multinodular and lobulated, and the stroma consisted of a mucus matrix with a reticulated growth of star- to spindle-shaped tumor cells (**Fig. 2A**–**2D**).

**Fig. 3:**
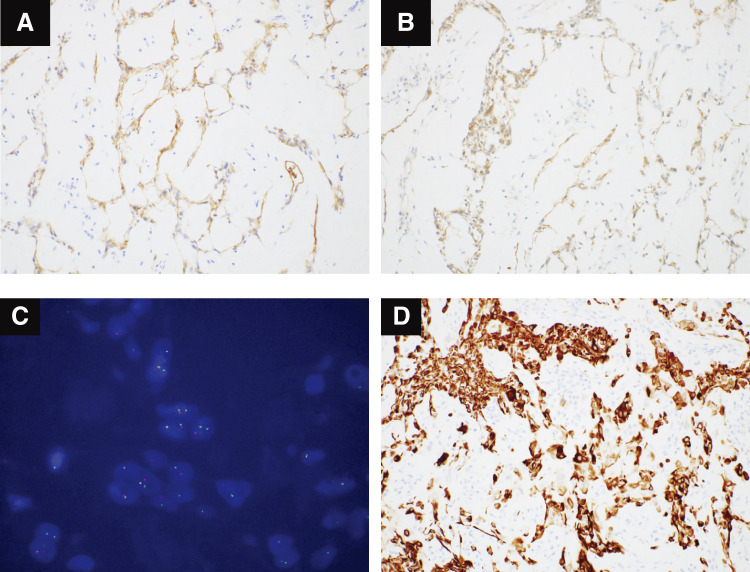
(**A**–**D**) Characteristic results of immunostaining and FISH analysis. (**A** and **B**) EMA and CD99 were positive (immunohistochemistry ×20). (**C**) FISH analysis showed that the split signal of EWSR1 was positive. (**D**) Desmin was strongly positive (usually negative for PPMS) (immunohistochemistry ×20). EMA, epithelial membrane antigen; EWSR1, Ewing sarcoma breakpoint region 1; FISH, fluorescence in situ hybridization

Immunostaining showed the tumor was negative for calretinin and D2-40, which completely ruled out mesothelioma, AE1/AE3 was partially positive, S100 was negative, smooth muscle actin (SMA) was focal positive, calponin was negative, epithelial membrane antigen (EMA) was weakly positive, CD68 was partially positive, and CD99 was positive. FISH analysis using the EWSR1 break-apart probe showed the split red and green signal, which suggested EWSR1 gene rearrangement. Thus, the tumor was diagnosed as PPMS (**Fig. 3A**–**3C**).

In addition, desmin was also strongly positive (**Fig. 3D**). This marker is frequently positive for myxoid angiomatoid fibrous histiocytoma (AFH), which some consider to be a lesion in the same spectrum as PPMS. Therefore, although morphologically PMS-like, this case is considered to be an overlap lesion of PPMS and AFH.^3)^

The patient underwent only surgical resection without adjuvant therapy and is alive 18 months postoperatively without recurrence.

## DISCUSSION

In this case, we initially suspected a chest wall tumor based on CT images, but the tumor was actually arising intrapulmonary. The tumor was different from typical PPMS in that it originated from the lung parenchyma without a bronchiolar component, and the immunostaining results indicated that it was an overlap lesion with AFH. **Table 1** shows a summary of PPMS cases reported in the literature so far. The frequency is gender-independent, and the size varies from 1 to 15 cm. In most cases, the lesions are located in the bronchi or bronchioles and sometimes infiltrate into the lung parenchyma. In some cases, however, the lesion originates in the peripheral lung, just below the pleura, which makes preoperative distinction from a chest wall tumor difficult.

**Table 1:** PPMS clinical data to date

Case No.	Age/Sex	Site	Size (cm)	FISH	RT-PCR	Treatment	Prognosis (months)
1^2)^	27/F	RUL	4.0	EWSR1	Pos	Surgery	NED (180)
2^2)^	33/F	LUL	3.5	EWSR1	Pos	Surgery	NED (144)
3^2)^	45/F	RUL	1.5	EWSR1	Neg	Surgery	NED (12)
4^2)^	36/F	L	NR	Neg	Neg	Surgery	DOD (brain meta. a few months)
5^2)^	32/F	RUL	NR	EWSR1	Pos	Surgery	NR
6^2)^	28/F	LLL	2.8	Neg	Pos	Surgery	Renal meta. → resection, NED (36)
7^2)^	67/M	LLL	2.8	EWSR1	Pos	Surgery	NR
8^2)^	68/F	RUL	2.0	Neg	Failure	Surgery	NR
9^2)^	63/F	LUL	NR	EWSR1	Pos	Surgery	NED (48)
10^2)^	51/M	RLL	2.0	EWSR1	Pos	Surgery	NR
11^9)^	31/M	LLL	2.7	NR	Pos	Surgery	NED (69)
12^4)^	26/M	LLL	9.0	EWSR1	Pos	Surgery	NED (19)
13^4)^	49/F	RLL	4.0	EWSR1	Pos	Surgery	NED (117)
14^4)^	54/F	RLL	4.5	EWSR1	Pos	Surgery	NED (152)
15^4)^	65/M	LLL	13.0	EWSR1	Pos	Surgery	Contralateral lung meta. (7) → resection, NED (72)
16^10)^	66/F	LUL	4.0	EW/CR	NR	Surgery	NED (18)
17^10)^	28/M	RLL	8.5	EWSR1	NR	Surgery	NED (16)
18^10)^	28/M	RUL	6.0	Neg	NR	Surgery	NED (4)
19^11)^	80/F	LLL	NR	EW/CR	NR	Endoscopic resection	Local reccurence (6) → Alive with disesase (36)
20^7)^	29/F	LLL	3.0	EWSR1	NR	Surgery	NED (17)
21^12)^	32/F	RML	2.6	EWSR1	Pos	Surgery	NED (96)
22^13)^	48/M	NR	>14.0	Neg	NR	CT → RT	Alive with disease (23)
23^14)^	61/F	RUL	1.8	NR	Pos	Surgery	NED (17)
24^15)^	21/F	PA	NR	EWSR1	Pos	Surgery	NED (38)
25^16)^	45/F	RUL	2.1	EWSR1	Pos	Surgery	NED (38)
26^8)^	37/M	LLL	2.3	EWSR1	NR	Surgery	NED (48)
27^5)^	64/F	RUL	5.5	EWSR1	Pos	Surgery	Pleural and bone meta. → alive with disease (24)
28^5)^	27/M	RLL	5.0	EWSR1	Pos	Surgery	NED (29)
29^5)^	45/M	LLL	3.0	EWSR1	Pos	Surgery	NED (24)
30^5)^	43/M	RLL	2.0	EWSR1	Pos	Surgery	NED (4)
31^5)^	23/M	RLL	3.0	EWSR1	Pos	Surgery	NED (3)
32^5)^	45/F	RUL	2.0	EWSR1	Pos	Surgery	NR
33^17)^	44/M	LUL	2.0	EWSR1	Neg	Surgery	NED (68)
34^18)^	64/M	RLL	15.0	NR	Pos	ND	Died after open biopsy (due to sepsis)
35^19)^	20/M	Left interlobar fissure	4.0	NR	Pos	Surgery	NED (30)
Our case	58/M	LUL	1.3	EWSR1	ND	Surgery	NED (18)

F, female; M, male; RUL, right upper lobe; LUL, left upper lobe; L, left; LLL, left lower lobe; RLL, right lower lobe; RML, right middle lobe; PA, pulmonary artery; EW/CR, EWSR1/CREB1; NR, not reported; Pos, positive; Neg, negative; ND, not done; NED, no evidence of disease; DOD, death of disease

Surgical resection was the treatment of choice in 34 of the 36 cases (the operative procedure varied from wedge resection to pneumonectomy depending on tumor size and location), while the remaining 2 cases were treated with endoscopic resection and chemoradiotherapy. Four of the 36 cases were reported to develop distant metastasis, including 1 case of brain metastasis that resulted in death.^2)^ In the remaining cases, 1 patient each had renal,^2)^ contralateral lung,^4)^ and pleural and bone metastases,^5)^ but they are still alive after resection of metastases or by just follow-up observation. Basically, the prognosis is very good if the tumor is completely resected. Even in cases with metastasis, a good prognosis is expected if the metastases are successfully resected.

A typical PPMS is an intrabronchial tumor with a lobular structure when observed under low magnification. A fibrous capsule is also often present. The tumor is usually composed of spindle-shaped, stellate, or polygonal cells that grow in a reticulate or cord-like fashion within a prominent mucinous stroma.^6)^ Histologically, the PPMS looks similar to other mucinous soft tissue and salivary gland tumors such as extraskeletal myxoid chondrosarcoma (EMC), myoepithelioma, and inflammatory myofibroblastomic tumor(IMT).^7)^ EMC was ruled out because he did not have a history of soft tissue tumors (it is extremely rare for EMC to arise intrathoracically as a primary), and the immunostaining showed that S100 was negative. Myoepithelioma was ruled out by the immunostaining results; myoepithelial markers, such as S100 and calponin, were negative. IMT is usually positive for SMA, desmin and anaplastic lymphoma kinase (ALK). However, this rare tumor does not have characteristic genetic fusions or mutations, which enabled us to rule out the differential diagnosis.

More rigorous differentiation from these tumors is possible based on the fact that PPMS is characterized by the EWSR1:CREB1 fusion. RT-PCR is recommended to confirm the gene fusion.^8)^ However, the fusion of EWSR1 with CREB1 itself has also been reported in other tumors such as hemangioma-like fibrous histiocytomas and soft tissue clear cell sarcomas, as described above. It is important to utilize all test results, including immunostaining markers and morphological features, to reach a more precise diagnosis.^4)^ Case No. 4 is the only case in which both FISH and RT-PCR were negative for mutations in the EWSR1 gene, and the patient died of brain metastasis a few months after resection of the primary lesion. The disease progression was clearly different from that of typical PPMS, and we cannot rule out the possibility that the patient had another tumor with histological similarity and with a known potential for distant metastasis, like EMC.^2)^ In cases where the diagnosis of PPMS cannot be strongly confirmed by each examination, more careful follow-up may be necessary.

In our case, although the characteristics of immunostaining, morphological features, and gene fusion were enough to reach the diagnosis of PPMS while ruling out other tumors as differentials, the strong positivity for desmin was atypical.^3)^ In the conventional literature, desmin, a protein that constitutes the cytoskeleton of myocytes, is usually negative in PPMS, while a higher percentage is positive in AFH, where myeloid stroma is considered a morphologic feature.

However, both PPMS and AFH have variations in histology, and these partially overlap. In addition to this, since they show the same gene fusion, some have suggested that they be considered as a single entity and given a single name to more accurately describe the lesions, rather than naming them individually as conspicuous patterns within a series of lesion variations.^3)^

We agree that these may be morphological variants of the same lesion and expect further discussion of this possibility in the future.

## CONCLUSIONS

At present, periodic follow-up and surgical resection when the tumor shows an increasing trend are the best and most realistic treatment for PPMS. However, some cases like ours have atypical findings for PPMS and are diagnosed as an overlap with another lesion, which makes it difficult to establish uniform criteria regarding malignancy, prognosis, and appropriate treatment.

## ACKNOWLEDGMENTS

The authors are sincerely grateful to Dr. Tomoyuki Shirase and Dr. Hiroshi Minato. Dr. Shirase, the chief of the Department of Pathology, Otsu Red Cross Hospital, first analyzed this lesion and decided to use the National Cancer Center Pathology Consultation Service to consult an expert for a rigorous diagnosis. In the service, a request is made to the online system provided by the Japanese Society of Pathology (Japanese Society of Pathology/National Cancer Center Pathology Diagnosis Consultation System). The consultation secretariat then requests cooperation from experts in cancer pathology diagnosis for each organ and area based on certain criteria. Dr. Minato, an expert of lung pathology and the chief of the Department of Pathology, Ishikawa Central Hospital, accepted the request from the secretariat and performed additional immunostaining and FISH analysis, leading to the diagnostic opinion of AFH/PPMS.

## DECLARATIONS

### Funding

Not applicable.

### Authors’ contributions

KH analyzed and interpreted the patient data and was a major contributor to writing the manuscript.

All authors have read and approved the final version of the manuscript.

All authors agree to be responsible for all aspects of the study.

### Availability of data and materials

The dataset supporting the findings of this study are available from the corresponding author upon reasonable request.

### Ethics approval and consent to participate

Not applicable.

### Consent for publication

The patient consented to reporting details of this case in a scientific publication.

### Competing interests

The authors declare that they have no competing interests.
